# 

**Published:** 1896-06

**Authors:** 


### CONTENTS:

### SELECTIONS.

Article I.—Anæsthesia, Local and General. By A. C. Hewitt, M. D. - - - - - - - - 49
" II.—Chronic Abscess. By Dr. M. L. Rhein, - - 61
" III.—Why are Our Teeth Degenerating, Arthur Turner, 65
" IV.—Preparation of Pulp-Cavity and Canals. By Dr. J. Foster Flagg, - - - - - - 67
" V.—Pulp Protection in Filling Teeth. By Dr J. D. Patterson, - - - - - - - 72
" VI.—Why are We Right-Handed, - - - - 75
" VII.—Local Anesthesia and Obtundents, - - - 78
" VIII.—Saw His Own Heart, - - - - - 82

### COMMUNICATED.

National Association of Dental Faculties, - - - - 85
Illinois State Dental Society, - - - - - - 86
The Chicago Dental Society, - - - - - - 86
The American Dental Society of Europe, - - - - 86
American Dental Association, - - - - - - 87
The National Association of Dental Examiners. - - - 87

### EDITORIAL, ETC.

Maryland Dental Law, - - - - - - - 88
Inter-State Medical Certificates. - - - - - - 91

### OBITUARY.

Dr. George S. Fouke. - - - - - - - 92

Dr. Felix E. Brown, - - - - - - - 93

### MONTHLY SUMMARY.

Hydrozone in Purulent Otitis Media, - - - - - 94
Peroxide of Hydrogen, - - - - - - - 96

